# Ecological and Functional Changes in the Hindgut Microbiome of Holstein Cows at High Altitudes

**DOI:** 10.3390/ani15020218

**Published:** 2025-01-15

**Authors:** Gong Chen, Haibo Lu, Shangzhen Huang, Congcong Zhang, Xiaojuan Ma, Bin Li, Lingling Hou, Qing Xu, Yachun Wang

**Affiliations:** 1 College of Life Sciences and Bioengineering, Beijing Jiaotong University, Beijing 100044, China; 22121620@bjtu.edu.cn (G.C.); 21121613@bjtu.edu.cn (C.Z.); 18636764982m@gmail.com (X.M.); llhou@bjtu.edu.cn (L.H.); 2 Laboratory of Animal Genetics, Breeding and Reproduction, Ministry of Agriculture of China, National Engineering Laboratory of Animal Breeding, College of Animal Science and Technology, China Agricultural University, Beijing 100193, China; luhaibo979@163.com (H.L.); huangshangzhen@cau.edu.cn (S.H.); 3 Institute of Animal Husbandry and Veterinary, Tibetan Academy of Agricultural and Animal Husbandry Sciences, Lhasa 850000, China; xukesuolibin@163.com

**Keywords:** hindgut microbiota, Qinghai–Tibetan Plateau, Holstein cows, environmental adaptation, 16S rRNA gene sequencing

## Abstract

The extreme environmental conditions of the Qinhai–Tibetan Plateau (QTP) challenge livestock survival and productivity, yet little is known about how high-altitude environments impact the gut microbiota of dairy cows. To fill this gap, we systematically investigated the differences in the hindgut microbiome between 87 plateau Holstein cows and 72 plain Holstein cows using 16S rRNA gene sequencing. Our analysis revealed that the hindgut microbiota of the plateau group exhibited lower species richness but higher evenness than that in the plain group. Additionally, significant separation in hindgut microbiota composition between the two groups was observed based on altitude, while parity, days in milk, and age did not show a comparable impact. Moreover, altitude had a lasting impact on bacterial communities and their co-occurrence networks, resulting in reduced microbial interactions and lower modularity in the plateau group. Furthermore, we identified four key microbial taxa, the *Bacteroidaceae* and *Rikenellaceae* families, as well as the *Prevotella* and *Treponema* genera, which were associated with the regulation of carbohydrate digestion and energy metabolism and might help the Holstein cows adapt to the plateau environment. Our findings provide insights into strategies for enhancing the adaptability of dairy cows to high-altitude environments through microbiota modulation, which could ultimately contribute to improving livestock management and sustainability in these extreme environments.

## Simple Summary

The gut microbiota is vital for maintaining physiological health and optimizing milk production in dairy cows, as well as dairy farm profitability, especially under the influence of high-altitude environments. However, limited information exists on how high-altitude environments affect gut microbiota, particularly in dairy cows, and has only recently gained attention. Therefore, this study aims to identify microbial differences and related pathways through sequencing data of gut microbiota from highland and lowland dairy cows. Our findings enhance our understanding of how the environment interacts with the gut microbiota to influence the physiological health of dairy cows. This might be critical for developing sustainable dairy cow breeding targets that emphasize high-altitude adaptability.

## 1. Introduction

The Qinghai–Tibetan Plateau (QTP), often referred to as ‘the roof of the world’, is the highest and largest plateau on Earth [1,2]. In QTP regions, dairy products are the traditional diet, and yak milk is the primary dairy source. In general, yaks exhibit relatively low production rates, with annual milk production lasting only 105–180 days and a daily output of only 2–4 kg [3]. In response to the increasing demand for dairy products, Holstein cows, renowned for their milk production, fat, and protein yields [4,5], have been introduced in QTP regions. However, the unique environmental conditions of the QTP, characterized by the low partial pressure of oxygen due to high altitudes [6], present significant challenges to the Holstein cows, even leading to death [7]. Additionally, intense UV radiation, sparse precipitation, and limited forage availability on high-altitude grasslands pose physiological challenges to Holstein cows [8,9,10]. These conditions constrain the expression of excellent traits, such as the milk production of Holstein cows. Moreover, previous studies reported that dairy cows exposed to high altitudes experience reduced body weight and metabolizable energy for milk production, leading to lower daily milk production compared to cows at lower altitudes [11,12].

As a key regulator of host physiology, the gut microbiota significantly influences various physiological functions, including digestion, energy harvesting, and immune regulation [13,14,15,16]. Millions of microbes live symbiotically within the host ruminant [17], playing a key role in converting plant material into energy [18,19] and increasing their tolerance to extreme environments [20]. As highlighted by several studies, the gut microbiota can adapt to environmental changes, with notable shifts occurring in response to various stressors, including altitude. This adaptation can occur within a few years, as the microbiota adjusts to the altered physiological conditions imposed by high-altitude environments [21,22]. Furthermore, studies have demonstrated that altitude impacts the structure of gut microbiota in mammals. For instance, altitude significantly influenced the composition of diet and gut microbial communities in plateau pikas [23]. Additionally, the rumen microbiome of *Ovis aries* (Tibetan sheep) and *Bos grunniens* (yaks) produced higher levels of volatile fatty acids, which aid these animals in adapting to the harsh conditions of high altitudes [24,25]. Additionally, differences in intestinal microbiota composition have been reported in yaks at varying altitudes, suggesting a dynamic microbiome response to altitude-related stressors [26]. In the case of dairy cattle, altitude-induced changes in microbiota composition have been shown to negatively impact the digestive efficiency and overall health of livestock. Specifically, in Sanhe heifers, where reduced digestive capability and health complications were observed at high altitudes [27]. Specifically, the genus *Prevotella*, which is often overrepresented in the microbiomes of high-altitude animals, plays a crucial role in carbohydrate fermentation. These changes were particularly associated with the production of short-chain fatty acids (SCFAs) from complex carbohydrates, a key metabolic process that may support the host’s adaptation to the low-oxygen environment by enhancing energy availability [28].

Despite these insights, limited studies have explored the specific effects of altitude on the intestinal microbiota of Holstein dairy cows, especially those introduced to high-altitude environments. Given the significance of microbiota in host adaptations and health, it is crucial to understand how environmental factors, such as altitude, influence the gut microbiome of dairy cattle, particularly in Holstein cows.

Therefore, this study aims to investigate the effect of high-altitude environments on the gut microbiome of Holstein cattle introduced to the Qinghai–Tibetan Plateau. By conducting differential analysis, pathway prediction analysis, and co-occurrence network analysis, we seek to elucidate the specific influences of a plateau environment on microorganisms. By exploring how these shifts influence the cows’ physiological adaptation, this research will provide valuable insights into potential microbiota-based strategies for improving the adaptability and production performance of dairy cattle through microbiota amendments.

## 2. Materials and Methods

### 2.1. Animal Selection and Fecal Sample Collection

**Figure 1:**
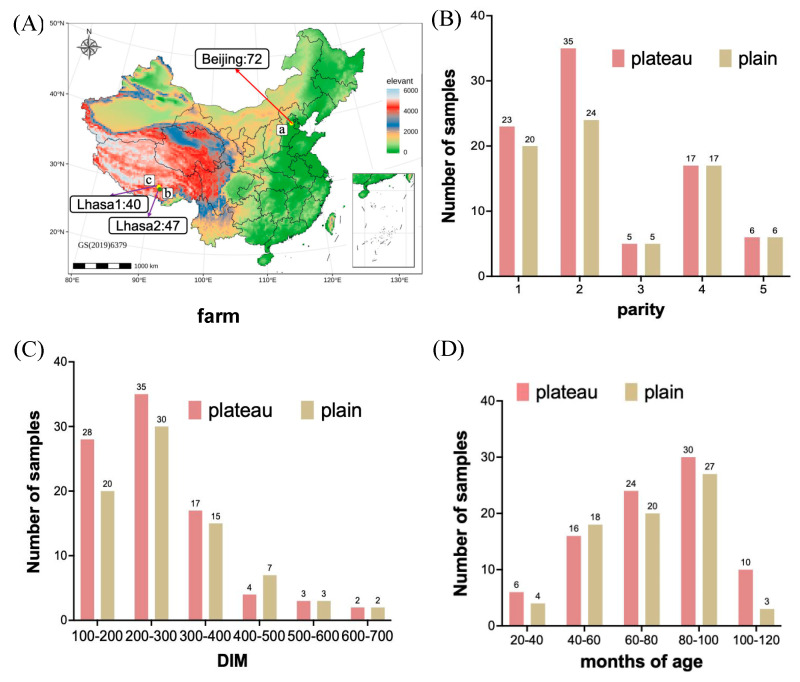
The characteristics of selected experimental animals. (**A**) a: seventy-two Holstein cows in the plain group from the Sanyuanluhe Treasure island in Beijing (latitude: 116.3503647; longitude: 39.54088527; altitude: 38 m), b: forty-seven in the plateau group from the Gaba ecological ranch in Lhasa (latitude: 91.24; longitude: 29.67; altitude: 3600 m), c: forty in the plateau group from the high-standard dairy cows breeding center in Lhasa (latitude: 91.26; longitude: 29.64; altitude: 3600 m). (**B**–**D**) Parities, DIM, and age in months of experimental animals.

Two groups of experimental animals were included in the current study: the plateau group (high altitude) and the plain group (low altitude). The plateau group consisted of 87 healthy Holstein cows from the high-standard dairy cows breeding center (40 animals) and the Gaba ecological ranch (47 animals) in Lhasa (altitude: 3600 m), China (Figure 1A). All 87 animals in the plateau group have lived in plateau areas for more than three years. The plain group included 72 healthy Holstein cows, of which 40 animals were selected in 2018 and 32 animals in 2019 at the Sanyuanluhe Treasure Island dairy farm located in Beijing (altitude: 38 m), China. The characteristics of selected Holstein cows are depicted in Figure 1B–D. The parity ranges from 1 to 5 in both plateau and plain conditions, and the first and second parity cows represent approximately 64% of the total samples, much more than other parity cows (Figure 1B). The days in milk (DIM) ranged from 100 to 700 days, with cows in the 100–300 DIM range being the most common, while those in the 400–700 DIM range were the least frequent (Figure 1C). The ages of the cows ranged from 20 to 120 months, with a significant proportion falling between 40 and 100 months (Figure 1D). As shown by the data, the distribution of the parity, DIM, and age in both the plateau and plain groups were relatively consistent, despite some variations. Fecal samples were manually collected from the rectum of the animals using sterilized gloves prior to morning feeding. The samples were then transferred to sterile 50 mL tubes, immediately quenched in liquid nitrogen, and stored at −80 °C for subsequent microbial analysis. All animals were fed the same diet of TMR at 0500, 1100, and 1700 h in the plateau and plain groups. Ad libitum water was available during the whole experiment. The TMR composition is shown in Table 1 below.

### 2.2. Microbial DNA Extraction and Sequencing

DNA was extracted from rumen fluid samples using the E.Z.N.A.^®^ Soil DNA Kit (Omega Bio-tek, Norcross, GA, USA) and assessed for concentration, purity, and quality using a spectrophotometer (NanoDrop 2000) and 1% agarose gel electrophoresis [29]. The V3–V4 hypervariable regions of the 16S rRNA genes were amplified with the primers 338F (5′-CCTAYGGGRBGCASCAG-3′) and 806R (5′-GGACTACHVGGGTWTCTAAT-3′) by a thermocycler PCR system (GeneAmp 9700, ABI, New York, NY, USA).

PCR reactions were extracted using the following protocol: initial denaturation at 95 °C for 3 min followed by 27 cycles of 95 °C for 30 s, annealing at 55 °C for 30 s, elongation at 72 °C for 45 s, and a final extension at 72 °C for 10 min. Each reaction, conducted in triplicate, consisted of a 20 μL mixture containing 4 μL of 5× FastPfu Buffer, 2 μL of 2.5 mM dNTPs, 0.8 μL of each primer (5 μM), 0.4 μL of FastPfu Polymerase, and 10 ng of template DNA. PCR products were extracted from a 2% agarose gel, purified with the AxyPrep DNA Gel Extraction Kit (Axygen Biosciences, Union City, CA, USA), and quantified using QuantiFluor™-ST (Promega, WI, USA) as per the manufacturer’s instructions. Purified amplicons were pooled in equimolar concentrations and sequenced using the paired-end (2 × 300 bp) protocol on an Illumina MiSeq platform (Illumina, San Diego, CA, USA) following standard procedures.

### 2.3. Quality Control of 16s Data

The Quantitative Insights Into Microbial Ecology (QIIME2, version 1.9.1) software was used to process the sequencing data [30]. Firstly, the tags and primer sequences in the original sequence were removed, and the original sequence was quality controlled, denoised, and spliced, and the chimera was removed by the QIIME2 deblur plugin [31,32]. The USEARCH [33] plugin in QIIME2 [34] was used to generate the operational classification unit (OTU) with a 97% sequence similarity as the threshold, and the species classification information was obtained by comparing it with the Greengenes database v13_8 (http://greengenes.microbio.me/, accessed on 19 November 2024) [35].

### 2.4. Species Community and Diversity Analysis

Based on the information on OTU and species abundance, Venn diagram analysis was carried out with the ggVennDiagram package in R language [36]. To analyze the difference in microbial richness and diversity in the hindgut of Holstein cows from the plateau and plain, α diversity was measured with the Ace, Chao1, and Shannon indexes. To identify differences in abundance in the hindgut microbiota between the plateau and plain groups, the β diversity was estimated by computing the unweighted Unifrac distance with the jackknifed_beta_diversity.py script (http://qiime.org/scripts/jackknifed_beta_diversity.html, accessed on 19 November 2024) in QIIME software (v2023.5). In β diversity analysis, PCoA (Principal Coordinate Analysis) was performed by the unweighted Unifrac distance [37]. Adonis was used to test the differences between groups and within groups [38]. Both α and β diversity analyses were carried out with the phyloseq, vegan, ggplot2, and ape packages in R language (version 4.4.1).

### 2.5. Co-Occurrence Network Analysis

Co-occurrence networks were constructed for different groups using the absolute abundance of OTUs. OTUs that occurred in at least 10% of all samples were considered for analysis, and relative abundance > 0.01% was selected for network analysis in the plateau and plain stages. We normalized the filtered OTU sequence counts for each microbial kingdom separately using the ’trimmed mean of M’ (TMM) method from the BioConductor package edgeR and expressed the normalized counts as relative abundance counts per million (CPM). The microbial network analyses were conducted to identify ecological clusters using the following protocol. Spearman’s correlation between any two OTUs was estimated, and only robust correlations with absolute correlation coefficients > 0.7 and FDR-adjusted *p*-values < 0.001 were used to construct networks using the ‘igraph’ package. The network was visualized using Hiplot (https://hiplot.com.cn/home/index.html, accessed on 19 November 2024) based on the R (v4.1.0) software packages “ggpubr”, “corrplot”, and “ggplot2” (The R Foundation for Statistical Computing). Network topology properties were calculated using the R package ‘igraph’.

### 2.6. LEfSe Analysis

The LEfSe method was performed on the website http://huttenhower.sph.harvard.edu/galaxy, accessed on 19 November 2024 [39]. Then, subjected to LDA, *p* < 0.05 (Kruskal–Wallis test) and the absolute LDA score (log10) ≥ 4 were considered significant differences in hindgut microbiota between the two groups.

### 2.7. Functional Prediction of Microbial Pathway Abundances by PICRUSt2

In order to obtain the function of intestinal microorganisms in different groups, Phylogenetic Investigation of Communities by Reconstruction of Unobserved States (PICRUSt2) software (v2.3.0) [40] and the KEGG database were used to predict the bacterial population and function. STAMP [41] was utilized to analyze the predicted metagenome and identify pathways linked to the resolution or persistence of altitude effects. The Benjamini–Hochberg method was applied to control the false discovery rate arising from multiple comparisons.

### 2.8. Statistical Analysis

Statistical analysis was performed using IBM SPSS 23.0 software, and graphs were generated using GraphPad Prism 7.0 (v5.03, GraphPad Software, San Diego, CA, USA). R version 4.4.1 was used for additional data analysis. Depending on the normality of the underlying data determined by the Shapiro–Wilk test, differences in alpha diversity were assessed by the Wilcoxon rank-sum test. Beta diversity was analyzed using Permutational Analysis of Variance (PERMANOVA) based on unweighted Unifrac dissimilarity, with significance determined via permutation tests. Differential abundance of microbial taxa was assessed using Linear Discriminant Analysis Effect Size (LEfSe) in R, and correlations between microbial communities and environmental variables were examined using Spearman’s rank correlation. The results were visualized with boxplots, bar charts, Principal Coordinates Analysis (PCoA) plots, and heatmaps. *p* < 0.05 was considered statistically significant.

## 3. Results

### 3.1. Hindgut Bacterial Community Diversity in OTU Richness Between the Plateau and Plain Holstein Cows

**Figure 2:**
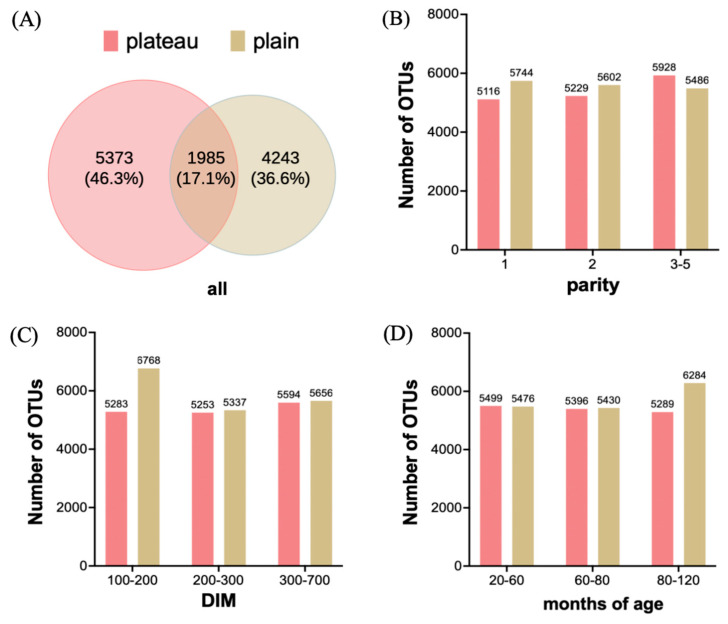
The number of OTUs detected in the plateau and plain Holstein cows. (**A**) OTU numbers of all cows in the plateau and plain groups; (**B**–**D**) OTU numbers of different parities, DIMs, and age in months of cows in the plateau and plain groups.

To examine microbial differences between the plateau group and the plain group, we conducted an OTU cluster analysis. A total of 9,273,171 raw sequence reads were obtained from the 159 samples. After splicing, quality control, and chimera filtration, a total of 5,480,431 high-quality sequences were generated, and the average number of sequences per sample was 34,468.12. Then, OTUs with an absolute abundance of less than 20 were removed. A Venn diagram (Figure 2A) demonstrated the unique and shared OTUs between two groups; 5373 and 4243 OTUs were unique in the plateau group and plain group, respectively, and 1985 OTUs, accounting for 17.1% of the total OTUs, overlapped both in the plateau group and plain group. The unique OTUs in the plateau group are more than those in the plain group, and the OTUs shared by the two groups are less than the unique ones. Also, we undertook a rigorous analysis by grouping and quantifying OTU numbers based on key parameters, including parity, DIM, and age in months. Parity was reclassified into subsets (1, 2, and 3–5) to ensure consistent individual counts within each category, as illustrated in Figure 1B. Similarly, DIM was segmented into intervals (100–200, 200–300, and 300–700) based on the data in Figure 1C, while age in months was segmented into 20–60, 60–80, and 80–120, as shown in Figure 1D. The results revealed that OTU numbers increased with parity in the plateau group but decreased in the plain group (Figure 2B). Fluctuations in OTUs were observed across different DIMs and age-in-month segments within both groups (Figure 2C,D). Specifically, the plateau group displayed a higher OTU count than the plain group in the 100–200 DIM (Figure 2C) and 80–120 age-in-month segments (Figure 2D).

### 3.2. Hindgut Bacterial Community Diversity in OTU Abundance Between the Plateau and Plain Holstein Cows

**Figure 3:**
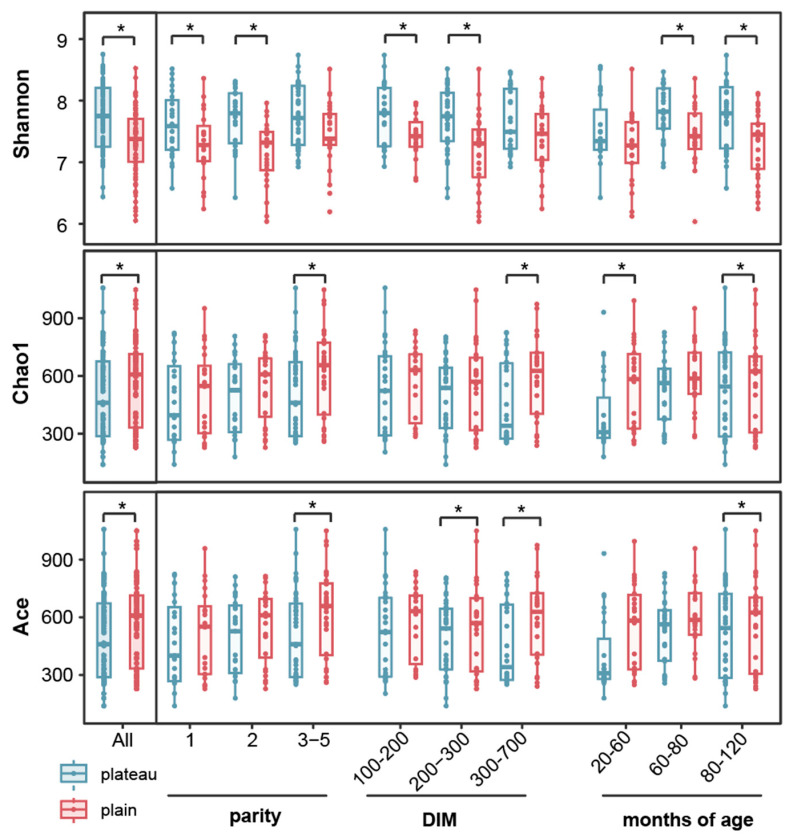
α diversity of microorganisms in plateau and plain Holstein cows. Shannon index, Chao1 index, and Ace index based on the abundance of OTUs. * Represents *p* < 0.05.

To characterize the hindgut bacteria between the plateau group and the plain group cows, DNA metabarcoding was used to target the bacterial species. For all samples, our analysis revealed significant differences in the Shannon, Chao1, and Ace diversity indices between the two groups (*p* < 0.05, respectively; Wilcoxon rank-sum test; Figure 3). Notably, the Shannon index was higher in the plateau group, while the Chao1 and Ace indices were higher in the plain group. Further examination unveiled significant distinctions in the Shannon and Chao1 indices for parities 1 and 2, as well as in the Chao1 and Ace indices for parity 3 (Figure 3, parity). Similarly, significant differences were observed in the Shannon index for the 100–200 DIM segment and in both Shannon and Ace indices for the 200–300 DIM segment. Additionally, differences in the Chao1 and Ace indices were noted for the 300–700 DIM segment (Figure 3, DIM). Also, significant variations were detected in the Chao1 index for the 20–60 month age group, the Shannon index for the 60–80 month age group, and all three indices (Shannon, Chao1, and Ace) for the 80–120 month age group (Figure 3, age in months).

**Figure 4:**
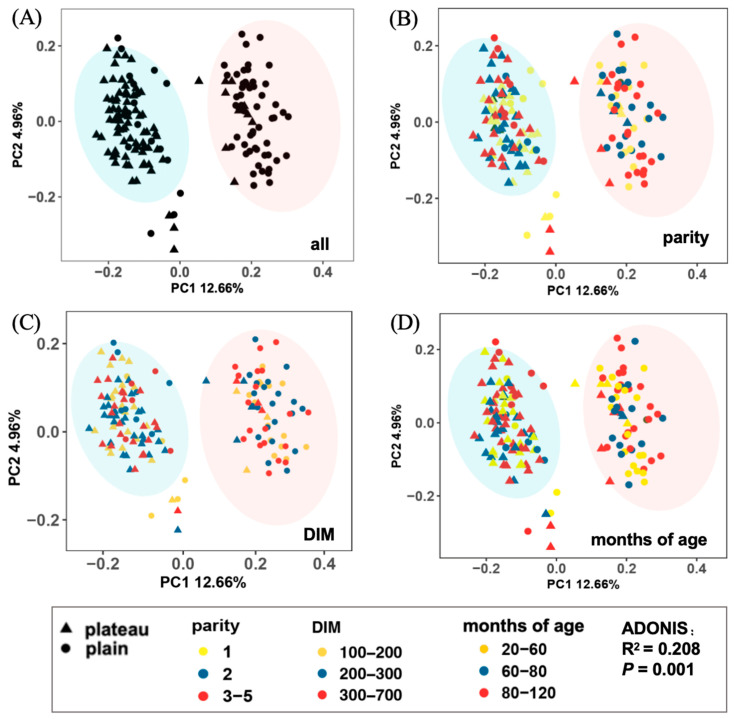
PCoA plot among microflora based on the abundance of OTUs in plateau and plain Holstein cows. (**A**) All cows in the plateau and plain groups. (**B**–**D**) Different parities, DIMs, and age in months of cows in the plateau and plain groups.

The comparison of β diversity using PCoA based on unweighted UniFrac distances revealed a clear clustering of samples according to management practices. Analysis of 18% of the variance explained by the first two axes of the PCoA for bacterial populations showed a significant association with management (Adonis analysis: R^2^ = 0.208, *p* = 0.001, Figure 4A). On the other hand, no significant separation was observed in the hindgut microbiota composition based on the different parities, DIMs, and age groups of the cows (Figure 4B–D). These findings suggested that the altitude, which serves as a proxy for management practices, had a more substantial impact on the intestinal microflora of dairy cows compared to variables such as parity, DIM, and age groups. Consequently, we decided to focus our follow-up analysis on the plateau and plain groups to further investigate the impact of altitude on the hindgut microbiome of cows.

### 3.3. Hindgut Bacterial Community Diversity in the Structure Between the Plateau and Plain Holstein Cows

**Figure 5:**
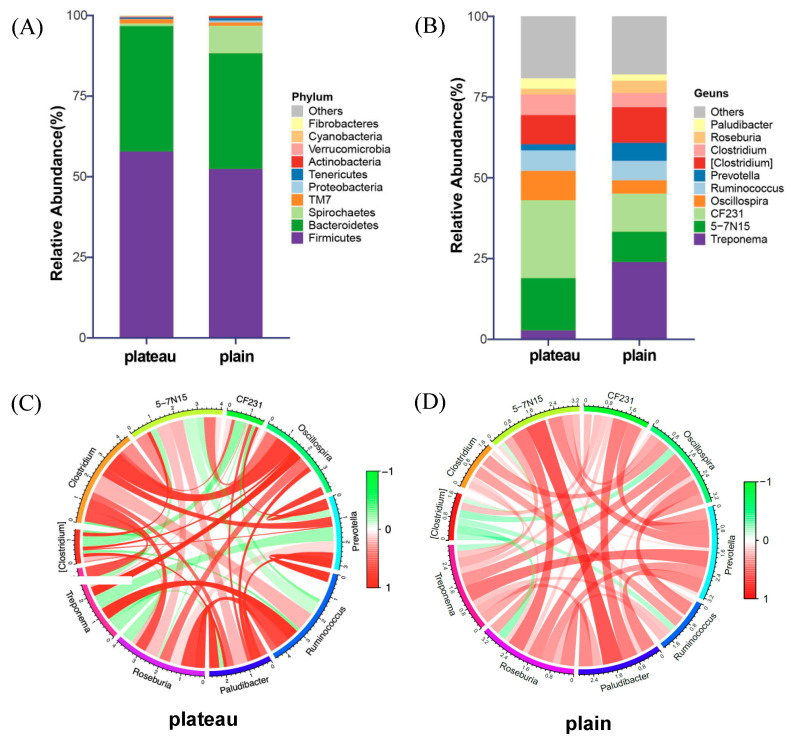
Relative abundances and correlation of hindgut bacteria between the plateau and plain Holstein cows. (**A**) Horizontal species composition accumulation chart for the top 10 phyla. (**B**) Horizontal species composition accumulation map of the top 10 genera. (**C**) Spearman correlation of the genus level in the plateau group. (**D**) Spearman correlation of the genus level in the plain group.

Statistics of the OTUs may demonstrate the relative abundance of the bacteria at the categorization of phylum, class, order, family, and genus. In the current study, a total of 12 bacterial phyla and 114 bacterial genera were identified. The most abundant phylum observed was *Firmicutes* (58.09%), followed by *Bacteroidetes* (33.69%), *Spirochaetes* (5.65%), *TM7* (0.86%), and *Proteobacteria* (0.51%), as shown in Figure 5A and detailed in Table 1. These five dominant phyla collectively represented over 98% of the total bacterial abundance. Among the top 10 abundant genera with average relative abundances exceeding 1%, key species included *5-7N15* (19.96%), *Treponema* (18.92%), *CF231* (17.56%), *Oscillospira* (8.75%), *Ruminococcus* (7.69%), *Clostridium* (6.98%), *[Clostridium]* (6.69%), *Prevotella* (6.25%), *Paludibacter* (3.91%), and *Roseburia* (3.62%) (refer to Figure 5B and Table 1). Notably, the abundance of *Treponema* and *Prevotella* in plateau Holstein cows was lower (3.74% and 2.78%) than in plain Holstein cows (34.10% and 9.72%). Detailed data on the relative abundance of these top 10 genera in the bacterial communities across the two groups are presented in Table 2.

To better understand how altitude gradient influenced the associations between hindgut bacteria in Holstein cows, we constructed relevant networks of the top 10 genera of bacteria in two groups. As shown in Figure 5C,D, most bacteria showed positive associations in both the plateau and plain groups, with stronger correlations observed within the plateau group. Notably, the genera *Treponema*, *5-7N15*, and *Prevotella* exhibited significant negative correlations in the plateau group, whereas in the plain group, these correlations were reversed. Similarly, *[Clostridium]* and *CF231* displayed contrasting correlation patterns between the two groups. These results suggested that the main bacterial taxa displayed varying correlations in the two groups, while consistency was observed in the relationships of other bacteria across both groups. A symbiotic relationship suggested a cooperative interaction that may enhance their growth and functional contributions to the microbial community, but the antagonism interaction implied competition or inhibition between the two genera. These contrasting interactions highlighted the influence of environmental conditions on microbial dynamics, as the favorable conditions in the plain group likely facilitated cooperative relationships, while the harsher or more competitive conditions in the plateau group may hinder such interactions.

### 3.4. Hindgut Bacterial Community Diversity in the Co-Occurrence Network Between the Plateau and Plain Holstein Cows

**Figure 6:**
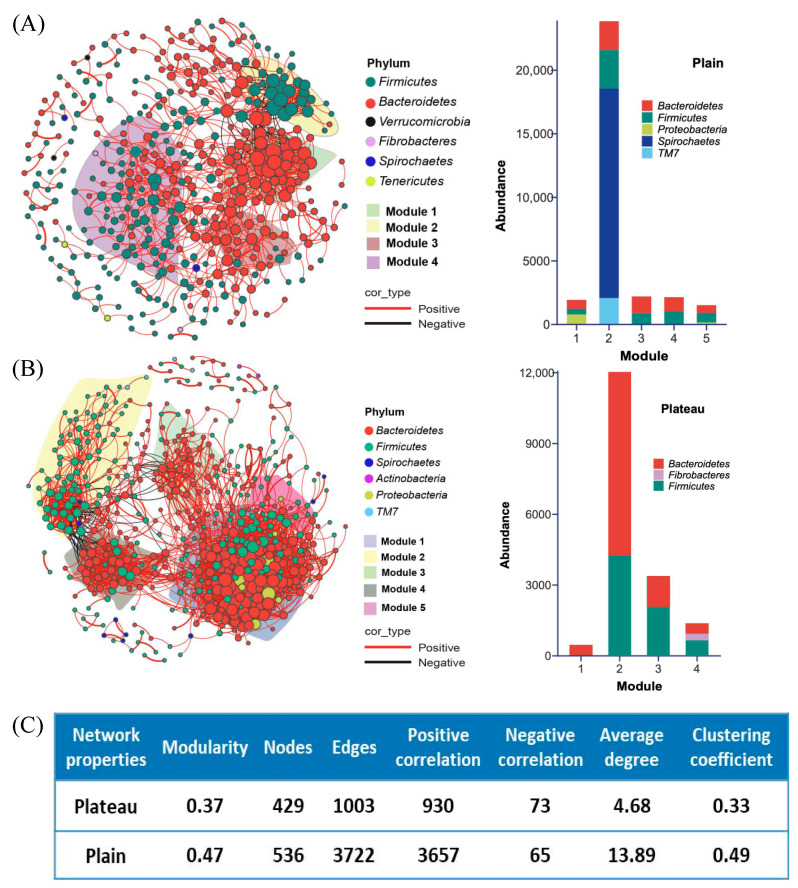
Co-occurrence network analysis for the bacterial community of plateau and plain Holstein cows. (**A**) The subnetworks for the abundant OTUs present in all samples of the plateau group and the distribution of major module classes in the associated networks. (**B**) The subnetworks for the abundant OTUs present in all samples of the plain group and the distribution of major module classes in the associated networks. (**C**) Co-occurrence network comparisons of the key topological parameters. The size of the points in the network diagram represent the size of the degree, the red lines between the points indicate a positive correlation, and the black lines are negative correlations.

Furthermore, the OTUs with a relative abundance of > 0.01% from plateau Holstein cows (1648) and plain Holstein cows (2011) were used to construct an ecological network of bacterial communities (FDR-adjusted *p*-value < 0.001, R > 0.7), with the results presented in Figure 6A,B. A total of 429 and 536 nodes, 1003 and 3722 edges, including 93% and 98% positive correlations of the ecological network, were obtained for plateau and plain group cows, respectively (Figure 6C). And, the betweenness_centrality of the network was 579 in the plateau and 696 in the plain group. The closeness_centrality of the network was 0.18 in the plateau and 0.07 in the plain group. The ecological network of the bacterial community in plain group cows had more links (with 13.89 average degrees) than in plateau group cows (with 4.68 average degrees and fewer nodes). On the other hand, the former (with a 0.47 modularity index and five modules) was better modularized than in plateau group cows (with a 0.37 modularity index and four modules, Figure 6C). Module analysis of the networks revealed four main modules in the plateau group (with a 0.33 clustering_coefficient) and five main modules in the plain group (with a 0.48 clustering_coefficient). *Firmicutes*, *Bacteroidetese*, *Verrucomicrobia*, *Fibrobacleres*, *Spirochaetes*, and *Tenericutes* dominated in all modules of an ecological network in plateau group cows (Figure 6A). For plain group cows, *Bacteroidetes*, *Firmicutes*, *Spirochaetes*, *Actinobacteria*, *Proteobacteria*, and *TM7* dominated in all modules (Figure 6B). So, the network observed in the plain group appeared more intricate and interconnected than the plateau group, indicating a higher level of interactions within the bacterial community, irrespective of taxonomic classification. Upon the OTUs representing microbial taxa in each node, distinct taxonomic profiles were observed. In the plateau group, the top four modules exhibited lower taxonomic diversity and were predominantly composed of *Bacteroidetes*, *Fibrobacteres*, and *Firmicutes*. While in the plain group, module2 displayed higher diversity and was dominated by *Bacteroidetes*, *Firmicutes*, *Spirochaetes*, and *TM7*, while module1, module3, module4, and module5 showed lower diversity with the dominance of *Bacteroidetes*, *Firmicutes*, or *Proteobacteria*. Also, the top three nodes (key nodes) exhibiting strong interactions with other nodes within different network modules in both the plateau and plain groups are listed in Table 3.

### 3.5. Different Bacteria Identified with the Relative Abundance of Hindgut Bacteria Between the Plateau and Plain Holstein Cows

**Figure 7:**
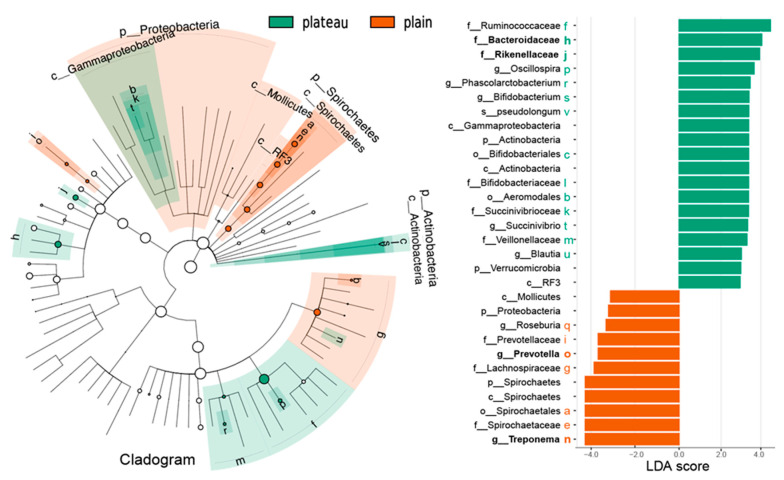
Different hindgut bacteria between the plateau and plain Holstein cows. The cladogram from the LEfSe analysis illustrated microbiome differences between the two groups across various phylogenetic levels. The concentric circles represent phylogenetic levels ranging from phylum to genus. Additionally, a distribution histogram based on LDA analysis was provided. The length of the histogram represents the LDA score. FDR< 0.05 and log10|LDA| > 2.0.

A linear discriminant analysis effect size (LEfSe) test was performed to assess the statistical differences in microbial communities between the plateau and plain groups. A total of 30 differentially abundant taxa with LDA > 2 were identified at six different taxonomic levels, from phylum to species, as illustrated in the cladogram presented in Figure 7. In the plateau group, three families stood out due to their relative abundance and significance: *Ruminococcaceae* (relative abundance > 27%, LDA > 4.0), *Bacteroidaceae* (relative abundance > 5%, LDA > 4.0), and *Rikenellaceae* (relative abundance > 3%, LDA > 3.5). Moreover, both *Bacteroidaceae* in the third module and *Rikenellaceae* in the first module were exclusive to the plateau group and ranked among the top thirty bacteria with strong interactions with other microorganisms, demonstrating degrees of interaction of 15 and 12, respectively, as shown in Figure 6A and detailed in Table 2 in co-occurrence network analysis.

Conversely, in the plain group, *Treponema* (relative abundance > 4%, LDA > 4.0), *Spirochaetaceae* (relative abundance > 4%, LDA > 4.0), and Spirochaetes (relative abundance > 4%, LDA > 4.0) showed increased abundance. Moreover, all of these taxa fall under the *Spirochaetes* phylum, highlighting their prominent presence in the plain group. Additionally, *Prevotella* also exhibited increased abundance in the plain group (as illustrated in the LDA score in Figure 7). Moreover, *Treponema* and *Prevotella* exhibited significant differences in the microbial community structure between the two environments, as highlighted in Result 2.4. In the plain group, *Treponema* comprised 34.1% of the microbial composition, while *Prevotella* accounted for 9.72%. In stark contrast, the plateau group displayed markedly lower proportions, with *Treponema* constituting only 3.74% and *Prevotella* 2.78% (see Table 1). Notably, the interactions between these two genera also varied between the two groups, as illustrated in Figure 5C,D. *Prevotella* also demonstrated a strong role in fostering interactions with other microorganisms, as evidenced by co-occurrence network analysis, where it was involved in the first module and achieved a degree of 81 (as shown in Table 2) in the plain group, but such interactions were not observed in the plateau group.

In summary, significant differences in microbial community composition between the plateau and plain groups were revealed, with distinct taxa exhibiting varying levels of abundance that likely contributed to the ecological dynamics of these environments. Specifically, *Bacteroidaceae* and *Rikenellaceae* in the plateau group, along with *Treponema* and *Prevotella* in the plain group, were identified as important taxa that play critical roles in shaping the microbial ecosystems within their respective environments.

### 3.6. Different Function Pathways in Hindgut Microbiota Between the Plateau and Plain Holstein Cows

**Figure 8:**
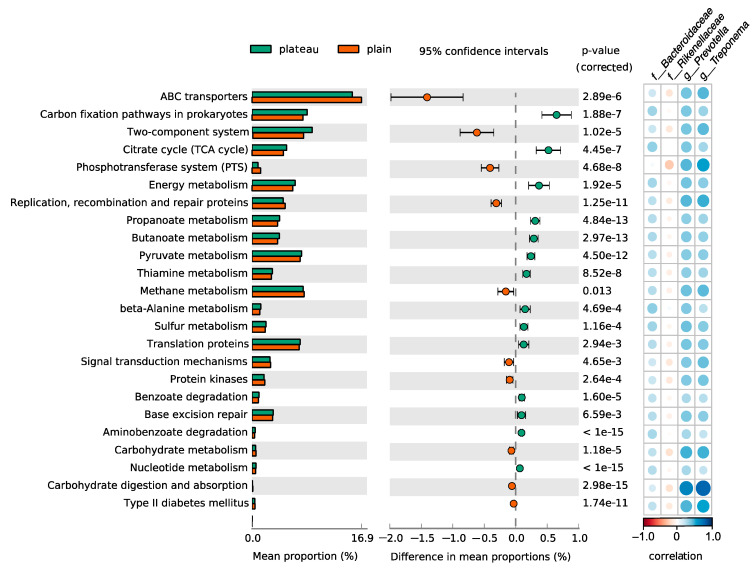
PICRUSt2-predicted KEGG function between the plateau and plain Holstein cows and correlation analysis with four key microorganisms. The map contains two parts. Error bar plots were generated to compare the two groups using Welch’s *t*-test on PICRUSt2-predicted KEGG function data. Bar graphs with extended error bars displayed only predicted functions with *p* < 0.05. The bar plots on the left represent the average proportion of each KEGG pathway, while the dotted plots on the right illustrate the differences in average proportions between the two groups (left part of the panel). Correlation analysis in four key bacterium and pathways. Blue designates a positive correlation while red designates a negative correlation (right part of the panel).

KEGG enrichment analysis was performed with 16s rRNA gene data to search the functional differences in hindgut microbiota between the plateau and plain Holstein cows. The PICRUSt2 analysis revealed four relevant classes (Organismal Systems, Metabolism, Environmental Information Processing, and Genetic Information Processing) comprising a total of 24 pathways at KEGG level 3 with significant differences (adjusted *p*-values < 0.05) between the two groups. Notably, 14 pathways were enriched in plateau Holstein cows, while 10 pathways showed an upregulation in the plain Holstein cows, with 15 of 24 pathways falling under the Metabolism class (see Figure 8). Specifically, the most three distinct pathways in the plateau group were the carbon fixation pathways in prokaryotes (mean proportions = 0.64, *p* = 1.88 × 10^−7^), the citrate cycle (TCA cycle, mean proportions = 0.51, *p* = 4.45 × 10^−7^), and energy metabolism (mean proportions = 0.36, *p* = 1.92 × 10^−5^), while ABC transporters (mean proportions = −1.40, *p* = 2.89 × 10^−6^), the two-component system (mean proportions = −0.61, *p* = 1.02 × 10^−5^), and the phosphotransferase system (PTS, mean proportions = −0.40, *p* = 4.68 × 10^−8^) were notably enriched in plain Holstein cows. Additionally, most of the 16 metabolic pathways were associated with carbohydrate metabolism, amino acid metabolism, and energy metabolism. Notably, 12 of the 16 metabolic pathways were upregulated in the plateau group. Taken together, these data indicated that the hindgut microbiota of plateau Holstein cows may possess distinct functional capabilities compared to their plain counterparts, potentially influencing their overall health and productivity. The microbial shifts resulting from the unique environmental conditions of the plateau likely impact the metabolic processes of Holstein cows. To further investigate the relationship between the four key microorganisms identified in Result 2.6 and the aforementioned pathways, a correlation analysis was conducted (Figure 8 right). The results revealed that *Bacteroidaceae*, *Prevotella*, and *Treponema* exhibited positive correlations with all pathways, while *Rikenellaceae* was negatively correlated with each of them. Notably, *Prevotella* and *Treponema* had extremely significant positive correlations with pathways related to carbohydrate metabolism, particularly carbohydrate digestion and absorption (R > 0.7).

## 4. Discussion

Due to specific factors in the Qinghai–Tibetan Plateau (QTP), such as low average annual temperatures, unpredictable climate variations, diverse vegetation, and high altitudes, there is currently a lack of comprehensive data on the gut microbiota of ruminants in this region [42]. This study systematically evaluated the differences in intestinal flora composition between 87 Holstein cows from the QTP and 72 Holstein cows from plain regions. The large sample size used in this analysis surpassed previous microbial studies in dairy cows, allowing for a more precise determination of microbial co-occurrence networks and other analyses. Additionally, the effects of altitude, parity, DIM, and age in months on the hindgut microbiota were examined through diversity analyses. By leveraging these results, we aimed to understand better how the unique environmental conditions of the QTP shaped the intestinal microflora of these ruminants.

The hindgut microbiota of dairy cows is affected by various factors, like different inflammation statuses [43], DIM [44], and host genetics [45]. To thoroughly investigate these effects, we first conducted a comprehensive analysis, grouping and quantifying OTU numbers based on key factors such as parity, DIM, and age in months. Our alpha and beta diversity analyses revealed that altitude had a more pronounced impact on the intestinal microflora of dairy cows than variables such as parity, DIM, and age. As a result, we focused our follow-up analysis on the plateau and plain groups to further elucidate the impact of altitude on the hindgut microbiome of cows. Interestingly, we found that the plateau group exhibited lower species richness but higher evenness than the plain group, which was consistent with the finding of a previous study that reported similar differences between high- and low-altitude yellow cattle [46]. This suggested that high altitudes significantly altered the structure of the hindgut microbiota in Holstein cows, potentially contributing to the occurrence and progression of plateau hypoxia.

Different microbial interactions within the hindgut microbiota were observed between the plateau and plain Holstein cows. Among the 12 bacterial phyla and 114 bacterial genera identified, *Firmicutes* (58.09%) and *Bacteroidetes* (33.69%) were the most abundant phyla (Figure 5A and Table 1) in both groups. Additionally, more than 95% of nodes in the microbial co-occurrence network belong to these two phyla (Figure 6A,B and Table 2). *Bacteroidetes* is known to aid in the digestion of complex carbohydrates [47], while *Firmicutes*, which consists of various fibrinolytic and cellulolytic bacterial genera, is the dominant species found in the gastrointestinal tract (GIT) of ruminants [48]. Both phyla have also been recognized as predominant in the rumen of plateau dairy cows [49]. Although no significant differences were detected in the relative abundances of *Bacteroidetes* and *Firmicutes* between the plateau and plain Holstein cows (Table 1), they occupied different modules within the co-occurrence network (Table 2), suggesting they may serve distinct roles by interacting with their specific partners in each group. The bacterial community in plain cows exhibited more complex yet concentrated microbial interactions than in plateau cows (Figure 6A,B). This intricate microbial network, characterized by high connectivity, supported microbial stability and enhanced responsiveness to environmental stressors such as drought, pollution, and hypoxia [50,51,52]. Thus, the high altitude and associated hypoxia reshaped the composition of hindgut microbiota and altered the diversity of intestinal microbes in plateau Holstein cows.

Several significantly different bacterial taxa at various taxonomic levels were revealed between the plateau and plain Holstein cows in this study. In conjunction with other analyses, four key microorganisms were highlighted as closely associated with the plateau environment. In the plateau group, the bacterial taxa of the *Bacteroidaceae* and *Rikenellaceae* families, both belonging to the Bacteroidetes phylum, exhibited higher abundance and demonstrated stronger interactions with other microorganisms within the associated networks (Table 2). However, while *Bacteroidaceae* showed a positive correlation with all functional pathways, *Rikenellaceae* exhibited the opposite trend (Figure 8). Previous studies have shown that the abundance of *Ruminococcaceae* serving as gut-beneficial bacteria is involved in carbohydrate degradation [53,54]. Moreover, *Bacteroidaceae* have also been shown to play a critical role in modulating host immune responses, particularly under environmental stressors, such as hypoxia and extreme temperatures, common in high-altitude regions [55]. Carbohydrates are known to influence the feed intake, intestinal methane, and milk yield in dairy cows [38]. This indicates that *Bacteroidaceae* may facilitate metabolic processes and enhance the overall functionality of the microbial community, contributing positively to the digestive efficiency and health of the cows in the plateau environment. In contrast, *Rikenellaceae* may have a limiting effect on these pathways, suggesting a more complex role that warrants further investigation.

In the plain group, the genera *Prevotella* and *Treponema* were enriched (Figure 7), with *Prevotella* showing significant correlations with other microorganisms within that group (Table 2). Moreover, *Prevotella* and *Treponema* were found to have a strong positive correlation with carbohydrate metabolism, as well as carbohydrate digestion and absorption (Figure 8). Consistent with our findings, the relative abundances of *Prevotella* and *Treponema* were reported to be lower at high altitudes in Sanhe cows or other cattle breeds [27]. A previous study has highlighted the reduced diversity of short-chain fatty acid (SCFA)-producing bacteria, including *Prevotella*, in ruminants from high-altitude environments, which may impact their overall metabolic efficiency [56]. *Prevotella* is known for producing SCFAs and is associated with improved glucose homeostasis, acting as a crucial contributor to microbial metabolites, including amino acids and carbohydrates involved in sucrose and galactose metabolisms [57], and it has been reported to positively correlate with milk protein yield [58]. *Treponema* is widely recognized as a lignocellulose-degrading bacterium and plays a key role in milk yield and acidogenesis in the rumen [59,60]. Moreover, a study by Zhang et al. (2024) emphasized the importance of *Treponemain* modulating the ruminal fermentation processes, which directly impacts milk yield and nutrient absorption in dairy cattle. The decline of these two key microorganisms in plateau Holstein cows may obstruct carbohydrate metabolism, adversely affecting the production performance and overall health of dairy cows in plateau environments.

## 5. Conclusions

This study provides the first comprehensive analysis of the hindgut microbiome in Holstein cows from the Qinghai–Tibetan Plateau (QTP), revealing significant differences in microbial composition and diversity between plateau and plain populations. The plateau group exhibited lower species richness but higher evenness, with altitude being a key factor driving the observed differences in microbial community structure. Notably, the hindgut microbiota of plateau Holstein cows showed reduced microbial interactions and lower modularity, which may reflect the adaptive challenges posed by the extreme high-altitude environment. Key microbial taxa identified in this study included the *Bacteroidaceae* and *Rikenellaceae* families, as well as the *Prevotella* and *Treponema* genera, which were identified as potentially critical in regulating carbohydrate digestion and energy metabolism, thereby adding the cows’ adaptation to fluctuating oxygen levels. In practical terms, feeding plateau dairy cows with feed containing specific microbes related to adaptation enhances their adaptation to the plateau and improves milk production. The lower carbohydrate metabolism induced by the hindgut microbiota in plateau Holstein cows may negatively affect the production and health of dairy cows in high-altitude environments. In light of these findings, targeted strategies to regulate the hindgut microbiota and enhance carbohydrate metabolism will hold significant promise for improving the welfare and overall health of cows in the plateau region in the future.

However, the current study primarily focuses on group-level differences, neglecting the impact of individual variation, such as parity, DIM, and months of age. Although our results suggest that altitude has a greater effect on the gut microbiome than these variables, their impact warrants further consideration. Additionally, this study lacks experimental approaches to explore the functional roles of specific microbes. The ruminal ecosystem is the real factor conditioning the ruminant’s digestive metabolism. In the future, we aim to integrate metagenomics, multi-omics, and longitudinal study designs focusing on rumen microbiota to overcome the current study’s limitations. Metagenomics will help explore the functional roles of microbes, while multi-omics integration will provide a comprehensive view of microbiome–host interactions. Longitudinal studies will allow us to track temporal changes and better understand microbial adaptation over time. These approaches will offer deeper insights into the microbiome’s role in highland adaptation.

## Figures and Tables

**Table 1:** Composition of the total mixed ration. ^1^ Per kilogram of premix contained the following: Ferric, 4500 mg; Copper, 1600 mg; Manganese, 3000 mg; Zinc, 5500 mg; Selenium, 30 mg; Cobalt, 20 mg; Iodine, 30 mg; vitamin A 600,000 IU; vitamin D 200,000 IU; vitamin E 200 IU.

Items	Content (%)
Corn silage	62.5
Wheat grass	20.83
Alfalfa hay	8.33
Oat	0.92
Wheat bran	0.75
Distillers’ dried grains with solubles	0.67
Corn	4.37
Soybean meal	0.5
Cottonseed meal	0.42
Premix ^1^	0.63
NaCl	0.08
Total	100

**Table 2:** Relative abundances of hindgut bacteria at the phylum and genus level between the plateau and plain Holstein cows.

Phylum				Genus			
Taxa	Plateau (%)	Plain (%)	Average (%)	Taxa	Plateau (%)	Plain (%)	Average (%)
*Firmicutes*	63.43	52.75	58.09	*5-7N15*	23.61	15.76	19.69
*Bacteroidetes*	33.59	33.79	33.69	*Treponema*	3.74	34.1	18.92
*Spirochaetes*	0.83	10.47	5.65	*CF231*	23.67	11.44	17.56
*TM7*	0.96	0.76	0.86	*Oscillospira*	12.56	4.94	8.75
*Proteobacteria*	0.06	0.96	0.51	*Ruminococcus*	9.52	5.85	7.69
*Tenericutes*	0.48	0.46	0.47	*Clostridium*	9.26	4.69	6.98
*Actinobacteria*	0.04	0.61	0.33	*[Clostridium]*	7.72	5.65	6.69
*Verrucomicrobia*	0.29	0.05	0.17	*Prevotella*	2.78	9.72	6.25
*Cyanobacteria*	0.19	0.08	0.14	*Paludibacter*	4.39	3.42	3.91
*Fibrobacteres*	0.12	0.07	0.1	*Roseburia*	2.79	4.44	3.62

**Table 3:** Each module moderately ranks the top thirty bacteria between the plateau and plain Holstein cows.

Module	Plateau			Plain		
	Phylum	OTU	Degree	Phylum	OTU	Degree
1	*Bacteroidetes*	*o_Bacteroidales*	16	*Bacteroidetes*	*g_5-7N15*	87
	*Bacteroidetes*	*f_Rikenellaceae*	12	*Bacteroidetes*	*g_Prevotella*	81
	*Bacteroidetes*	*g_5-7N15*	7	*Proteobacteria*	*g_Succinivibrio*	75
2	*Firmicutes*	*g_[Clostridium]*	27	*Firmicutes*	*f_Ruminococcaceae*	28
	*Bacteroidetes*	*f_p-2534-18B5*	22	*Bacteroidetes*	*f_S24-7*	19
	*Firmicutes*	*f_Ruminococcaceae*	20	*Bacteroidetes*	*g_CF231*	16
3	*Bacteroidetes*	*g_5-7N15*	32	*Bacteroidetes*	*o_Bacteroidales*	27
	*Bacteroidetes*	*f_Bacteroidaceae*	15	*Bacteroidetes*	*f_S24-7*	15
	*Bacteroidetes*	*o_Bacteroidales*	13	*Firmicutes*	*f_Ruminococcaceae*	3
4	*Firmicutes*	*f_Ruminococcaceae*	18	*Firmicutes*	*g_Phascolarctobacterium*	33
	*Bacteroidetes*	*o_Bacteroidales*	9	*Bacteroidetes*	*g_RF16*	31
	*Firmicutes*	*o_Clostridiales*	9	*Bacteroidetes*	*g_CF231*	29
5				*Firmicutes*	*f_Ruminococcaceae*	42
				*Firmicutes*	*f_Lachnospiraceae*	25
				*Firmicutes*	*g_Oscillospira*	19

## Data Availability

The original contributions presented in the study are included in the article, further inquiries can be directed to the corresponding author.
